# Supplementary material for: MetaRibo-Seq measures translation in microbiomes
Source: Nat Commun. 2020 Jun 29;11:3268. doi: 10.1038/s41467-020-17081-z (PMC7324362; doi:10.1038/s41467-020-17081-z)
Supplement: Supplementary file 10 — Supplementary Data 7 [file 41467_2020_17081_MOESM10_ESM.zip › File2/Confidence_High_Taxonomy/222550_out.krona.html]

Javascript must be enabled to view this page.

members
magnitude
magnitudeUnassigned
count
unassigned
taxon
rank

222550\_out

86

2
85
superkingdom

phylum
1239
85

class
85
186801

186802
85
order

65
541000
family

64
genus

SRS022137\_contig\_number\_contig-100\_27698.70044SRS1041091\_contig\_number\_contig-100\_32381.67813
216851
2

species
2

SRS022609\_contig\_number\_10744SRS024663\_contig\_number\_13953
1971605


SRS013687\_contig\_number\_contig-100\_1669.185504SRS013965\_contig\_number\_37778SRS014855\_contig\_number\_contig-100\_471.42285SRS015694\_contig\_number\_24339SRS015854\_contig\_number\_14021SRS016056\_contig\_number\_contig-100\_8930.8930SRS016335\_contig\_number\_contig-100\_3802.252307SRS018575\_contig\_number\_contig-100\_21462.21462SRS018623\_contig\_number\_contig-100\_3473.119563SRS018817\_contig\_number\_23373SRS018984\_contig\_number\_6318SRS019496\_contig\_number\_16798SRS019693\_contig\_number\_9156SRS020233\_contig\_number\_52424SRS020869\_contig\_number\_37722SRS023526\_contig\_number\_34424SRS023914\_contig\_number\_24230SRS024075\_contig\_number\_16561SRS024132\_contig\_number\_contig-100\_9065.213641SRS024492\_contig\_number\_15037SRS049446\_contig\_number\_14933SRS049959\_contig\_number\_36171SRS050752\_contig\_number\_contig-100\_1772.87328SRS050941\_contig\_number\_16389SRS052027\_contig\_number\_18756SRS052697\_contig\_number\_15375SRS062654\_contig\_number\_contig-100\_3103.158063SRS063040\_contig\_number\_contig-100\_7476.203135SRS076804\_contig\_number\_13889SRS076976\_contig\_number\_3943SRS077849\_contig\_number\_contig-100\_3735.184326SRS097889\_contig\_number\_14402SRS098073\_contig\_number\_17701SRS098644\_contig\_number\_14075SRS098881\_contig\_number\_16072SRS101376\_contig\_number\_contig-100\_5160.140094SRS104084\_contig\_number\_6044SRS1041039\_contig\_number\_24793SRS104311\_contig\_number\_38412SRS104693\_contig\_number\_364SRS1055056\_contig\_number\_5332SRS142503\_contig\_number\_16166SRS142890\_contig\_number\_27747SRS142980\_contig\_number\_2680SRS143342\_contig\_number\_contig-100\_10889.88090SRS143372\_contig\_number\_9214SRS143417\_contig\_number\_936SRS143523\_contig\_number\_contig-100\_9214.115991SRS143780\_contig\_number\_13763SRS144135\_contig\_number\_13121SRS144362\_contig\_number\_34449SRS144537\_contig\_number\_contig-100\_4338.219079SRS148424\_contig\_number\_36171SRS149075\_contig\_number\_179SRS149181\_contig\_number\_contig-100\_1191.193188SRS149325\_contig\_number\_15540SRS893259\_contig\_number\_6027SRS893270\_contig\_number\_21853SRS893288\_contig\_number\_3605SRS893378\_contig\_number\_contig-100\_2178.129553
853
60
species

genus
1263
1

1
1637508

SRS1041145\_contig\_number\_contig-100\_615.616
species

species
1897045

SRS015431\_contig\_number\_84309SRS015782\_contig\_number\_14664SRS017103\_contig\_number\_11902SRS019030\_contig\_number\_contig-100\_24867.68474SRS019068\_contig\_number\_contig-100\_3610.305128SRS021484\_contig\_number\_contig-100\_3152.172056SRS045739\_contig\_number\_contig-100\_5276.70347SRS047044\_contig\_number\_contig-100\_4572.4572SRS054905\_contig\_number\_contig-100\_4042.131747SRS055017\_contig\_number\_34048SRS056273\_contig\_number\_contig-100\_4053.207413SRS075078\_contig\_number\_13509SRS098061\_contig\_number\_contig-100\_3684.83646SRS104400\_contig\_number\_contig-100\_3882.221494SRS143181\_contig\_number\_contig-100\_4040.81376SRS143466\_contig\_number\_contig-100\_12960.110662SRS143991\_contig\_number\_contig-100\_1640.176956SRS146812\_contig\_number\_14708SRS147614\_contig\_number\_contig-100\_4724.4725SRS149784\_contig\_number\_contig-100\_9822.95958
20

1

SRS1041137\_contig\_number\_contig-100\_35834.35835
